# VENUS, a Novel Selection Approach to Improve the Accuracy of Neoantigens’ Prediction

**DOI:** 10.3390/vaccines9080880

**Published:** 2021-08-09

**Authors:** Guido Leoni, Anna Morena D’Alise, Fabio Giovanni Tucci, Elisa Micarelli, Irene Garzia, Maria De Lucia, Francesca Langone, Linda Nocchi, Gabriella Cotugno, Rosa Bartolomeo, Giuseppina Romano, Simona Allocca, Fulvia Troise, Alfredo Nicosia, Armin Lahm, Elisa Scarselli

**Affiliations:** 1 Nouscom S.R.L., Via di Castel Romano, 00100 Rome, Italy; m.dalise@nouscom.com (A.M.D.); fg.tucci@nouscom.com (F.G.T.); e.micarelli@nouscom.com (E.M.); i.garzia@nouscom.com (I.G.); m.delucia@nouscom.com (M.D.L.); f.langone@nouscom.com (F.L.); l.nocchi@nouscom.com (L.N.); g.cotugno@nouscom.com (G.C.); r.bartolomeo@nouscom.com (R.B.); g.romano@nouscom.com (G.R.); s.allocca@nouscom.com (S.A.); f.troise@nouscom.com (F.T.); a.lahm@nouscom.com (A.L.); e.scarselli@nouscom.com (E.S.); 2 Department of Molecular Medicine and Medical Biotechnology, University of Naples Federico II, Via Federico Pansini, 80131 Naples, Italy; alfredo.nicosia@unina.it; 3 CEINGE, Via Comunale Margherita, 80131 Naples, Italy

**Keywords:** neoantigen, cancer vaccine, VENUS, prediction, MC38

## Abstract

Neoantigens are tumor-specific antigens able to induce T-cell responses, generated by mutations in protein-coding regions of expressed genes. Previous studies demonstrated that only a limited subset of mutations generates neoantigens in microsatellite stable tumors. We developed a method, called VENUS (Vaccine-Encoded Neoantigens Unrestricted Selection), to prioritize mutated peptides with high potential to be neoantigens. Our method assigns to each mutation a weighted score that combines the mutation allelic frequency, the abundance of the transcript coding for the mutation, and the likelihood to bind the patient’s class-I major histocompatibility complex alleles. By ranking mutated peptides encoded by mutations detected in nine cancer patients, VENUS was able to select in the top 60 ranked peptides, the 95% of neoantigens experimentally validated including both CD8 and CD4 T cell specificities. VENUS was evaluated in a murine model in the context of vaccination with an adeno vector encoding the top ranked mutations prioritized in the MC38 cell line. Efficacy studies demonstrated anti tumoral activity of the vaccine when used in combination with checkpoint inhibitors. The results obtained highlight the importance of a combined scoring system taking into account multiple features of each tumor mutation to improve the accuracy of neoantigen prediction.

## 1. Introduction

The development of therapeutic vaccines for the treatment of solid tumors had only limited success in the past, likely because vaccine-induced T-cells are inhibited by several immunosuppressive mechanisms activated in tumor microenvironment [1]. More recently, the success of immunotherapy based on immune checkpoint inhibitors has opened the opportunity to revisit therapeutic vaccines based on tumor antigens as a potentially complementary mechanism of action to improve clinical efficacy when delivered in combination with anti-PD-1 or other checkpoint inhibitors (CPIs) [2,3].

Tumor antigens can be classified into different categories: self-antigens, tissue differentiation antigens, and neoantigens derived from mutated self-proteins. Whether and to what extent spontaneous immune responses against self-antigens have an impact on tumor growth is still being evaluated [4]. In contrast, there is compelling evidence that supports the concept that neoantigens represent a promising target for cancer vaccination [5]. Cancer neoantigens are present exclusively in tumor cells and not in normal cells and have been shown to play a significant role in recognition and killing of tumor cells by CD8 and CD4 T-cell mediated immune responses [6].

The advent of next generation sequencing (NGS) has unveiled the mutational spectra of human tumors highlighting the fact that the total number of somatic mutations may vary considerably with tumor histology and from patient to patient. The results obtained indicate single nucleotide variants (SNVs) as the major source of mutations generating neoantigens, with insertion/deletion mutations (indels) generating a frame shift peptide (FSP) being a second source, though less frequent [7]. However, indels, different from SNVs, encode in many cases a relatively long neo-antigenic amino acid sequence and are expected to be particularly immunogenic because of their reduced similarity with “self” proteins [8], even if relatively rare FSPs should be considered in addition to SNVs, for vaccine design.

Several studies have provided clear evidence that, for mismatch repair machinery proficient tumor types, very few somatic mutations are shared among patients [2,3]. Therefore, the development of therapeutic cancer vaccines requires a personalized analysis of each patient tumor. Importantly, such an analysis often results in the identification of hundreds of tumor-specific mutations; however, only few of them are actually immunogenic neoantigens and can induce T cells capable of recognizing the tumor. For some mutations, the corresponding potential neoantigens may not be recognized by the immune system because their neo-epitopes are not processed or presented by the tumor cells or because immune tolerance mechanisms led to the elimination of T cells reactive against the mutated neo-peptides later occurring when they are too similar to the wild type counterpart [9].

Most currently tested cancer vaccine platforms have limited capacity to deliver a large number of neoantigens, making it impossible to target all or even most of the tumor specific mutations, thus raising the need for an efficient method to select the most suitable mutated peptides to be targeted by a vaccine [10]. Although several neoantigen prioritization methods have been published, the prediction of effective neoantigens that are mutated peptides displayed on the tumor cell and recognized by the immune system is still a challenging task.

Many of the current prioritization methods apply fixed thresholds on the likelihood of binding to class I major histocompatibility complex (MHC), although experimental data support the notion that effective immunogenic neoantigens cover a broad range of predicted affinities for a patient’s MHC alleles [11]. Moreover, estimating the abundance of a neoantigen in a tumor sample needs to accounts not only for the number of tumor cells that carry the mutation, but also the expression levels of mRNA transcripts of the gene and, more specifically, of the gene region comprising the mutation. Therefore, it is crucial to develop prioritization methods that select neoantigens avoiding the limitations of current methods.

We have developed a scoring method, called VENUS (vaccine-encoded neoantigens unrestricted selection), to prioritize mutated peptides with a high likelihood of inducing an immune response effective against tumor cells. We have validated our prioritization method for its ability to include among the top selected, those that have been experimentally shown to induce a spontaneous immune response in human subjects.

The method of neoantigen selection was also validated using a tumor mouse model in which we demonstrated the therapeutic activity of an adeno viral vector vaccine encoding the top scored neoantigens identified by VENUS.

## 2. Materials and Methods

### 2.1. Analysis of NGS Data from Patients with Solid Tumours

Publicly available Exomeseq and RNAseq data from nine patients with different solid tumors were downloaded from the SRA database (Bioproject IDs: PRJNA298330; PRJNA298310; PRJNA298376). A preliminary quality control of the raw sequence data was performed by filtering out reads of low quality with Trimmomatic-0.33 [12] (LEADING: 5; TRAILING: 5; SLIDINGWINDOW: 4:20; MINLEN: 50). The remaining DNA and RNA read pairs were then aligned against the human reference genome version GRCh38/hg38 using BWA-mem [13] and hisat2 [14], respectively. Read pairs for which only one read is mapped and paired reads that align to more than one genomic locus with the same mapping score were filtered out. Exomeseq alignments were then further processed by optimizing the local alignment around small indels, marking duplicated reads and recalibrating the final base quality score in the realigned regions (program, parameters) with Picard tools (http://broadinstitute.github.io/picard/ (v2.20 accessed on September 2019). Somatic variant calling of SNVs and indels was performed on the recalibrated DNA read data using mutect2, Varscan2, and SCALPEL by explicitly comparing the tumor DNA data versus the normal (blood) control DNA data [15,16,17]. Only SNVs that generate a non-synonymous (missense) change within a codon or indels that generate a change of the reading frame within the coding sequence of protein-coding genes, identified by at least one variant caller, were retained. The initial list of SNVs and frame shift indels was then further reduced by selecting only mutations that fulfil the following criteria:

- mutation allele frequency (MF) in the tumor DNA sample ≥ 10%;
- ratio of the MF in the tumor DNA sample and in the control DNA sample ≥ 5;
- number of mutated reads at chromosomal position of somatic variant in the tumor DNA > 2;
- number of mutated reads at chromosomal position of somatic variant in the normal DNA < 2.

Each somatic variant is then translated into a peptide containing the mutated amino acid in order to predict binding of the putative neoantigen to the patient’s MHC class-I alleles. For missense SNVs, the neoantigen peptides are generated as 25 mer peptides with the mutated amino acid flanked upstream and downstream by 12 wild type (wt) amino acids. For indels generating frame shift, the FSP starts with the first mutated amino acid generated by the insertion/deletion and extends downstream up to the first encountered stop codon. All mutations generating a FSP of at least 1 aa in length are retained. A modified FSP is then generated by addition of 12 wild type amino acids upstream to the first new aa. Only modified FSPs that have a final length of at least nine amino acids are retained.

To avoid the inclusion of FSP sequence stretches with a low probability of being presented by MHC Class-I, a “tailoring” procedure is then applied (Supplementary Figure S1) taking into account the MHC Class-I binding IC50 values for all possible 9-mer epitopes within the complete modified FSP. Each 9-mer epitope is then extended into a 25 mer by adding eight amino acids to both ends. All FSP-derived 25 mers are then added to the list of SNV-derived 25 mer and subjected to the ranking procedure.

Gene expression estimates were performed using the Rsubsreads package [18] and converting the raw counts estimated on refseq annotated genes, in transcripts for million (TPM).

HLA class-I binding prediction is based on patient-specific HLA class-I type assessment performed by aligning the QC-filtered DNA reads from the healthy sample on the portion of hg38 genome that encodes the class-I human haplotypes with BWA-mem. Read pairs for which only one read is aligned and read pairs aligned to more than one locus with the same mapping score are filtered out using Samtools 1.4 [19]. Finally, determination of the patient’s most likely haplotypes is performed with Optitype [20]. MHC-I binding predictions for 25 mers are performed using the consensus method of the IEDB 2.17 software considering only predicted epitopes comprising a non-wt amino acid [21].

### 2.2. Exome and RNA Sequencing of Mice Tumors

DNA and RNA library construction and NGS of tumor samples were performed at CeGaT GBMH (Tubingen, Germany). Genomic DNA was fragmented and used for Illumina library construction. Exonic regions were captured in solution using the Agilent mouse Sure Select All Exon kit 50 Mb. Paired-end sequencing (2 × 100 bp) was performed with the Hiseq2000 Genome Analyser (Illumina, San Diego, California) at a target coverage of 120×. RNA was fragmented and the sequencing library was prepared using Illumina TruSeq mRNA stranded kit. Sequencing was performed with the Hiseq2000 Genome Analyser (Illumina) at a target depth of 60 million read pairs (2 × 100 bp). Germline sequence data of the C57/bl6 murine strain were downloaded from SRA (experiment id: SRX089130) and used as normal control sample. Raw NGS data were aligned on mm10 genome. The analysis of mouse NGS was performed with the same protocol used for the human samples.

### 2.3. VENUS RSUM Score

The VENUS RSUM score is the weighted sum of individual ranks obtained by sorting the list of detected neo-peptide derived 25 mers according to three independent parameters.

(1) RSUM=(RFREQ+REXPR+(k+RIC50))∗WF

RFREQ corresponds to the minor allele frequency of the mutation present in the 25mer neo-peptide.

REXPR is the expression level of a mutation estimated from the tumor RNAseq data as starting from gene-centered TPM value. This TPM value is then modified taking into account the number of mutated and wild type reads detected in the fragment containing the mutation by the RNAseq transcriptome data and called corrected TPM (corrTPM) (2):

(2) $$\mathrm{corrTPM}=\mathrm{TPM}\left(\mathrm{gene}\right)*\left(\frac{\mathrm{num}\;\mathrm{reads}\;\left(\mathrm{mut}\right)+0.1}{\mathrm{num}\;\mathrm{reads}\;\left(\mathrm{mut}\right)+\mathrm{num}\;\mathrm{reads}\;\left(\mathrm{wt}\right)+0.1}\right)$$

RIC50 is the predicted likelihood of binding to MHC-I as estimated by the IEDB software.

k is a constant value (penalty) that is added to the RIC50 value in case the predicted epitope has an IC50 value higher than 1000 nM (3).

(3) $$k=\{\begin{array}{ccc} & \mathrm{number}\;\mathrm{of}\;\mathrm{candidate}\;\mathrm{neoantigens} & {\mathrm{if}\;\mathrm{MHCI}}_{\mathrm{IC}50\;\mathrm{prediction}}>\;1000\;\mathrm{nM}\\ 0 & & {\mathrm{if}\;\mathrm{MHCI}}_{\mathrm{IC}50\;\mathrm{prediction}}\leq C50\;\mathrm{pre} \end{array}$$

Finally, a down-weighting factor (WF) is introduced according to the scheme provided below to penalize cases where, in general for technical reasons, the RNAseq data do not provide coverage at the location of the mutation, neither for the wild type aa (wt control reads) nor for the mutated aa (tumor reads) in an otherwise expressed gene.

(4) $$\mathrm{WF}=\{\begin{array}{ll} 1\;\; & \mathrm{mut}\;\mathrm{reads}\;\mathrm{RNAseq}>0\\ 2\;\; & \mathrm{mut}\;\mathrm{reads}\;\mathrm{RNAseq}=0;\mathrm{wt}\;\mathrm{reads}\;\mathrm{RNAseq}=0;\mathrm{TPM}\geq 0.50\\ 3\;\; & \mathrm{mut}\;\mathrm{reads}\;\mathrm{RNAseq}=0;\mathrm{wt}\;\mathrm{reads}\;\mathrm{RNAseq}>0;\mathrm{TPM}\geq 0.50\\ 4\;\; & \mathrm{mut}\;\mathrm{reads}\;\mathrm{RNAseq}=0;\mathrm{wt}\;\mathrm{reads}\;\mathrm{RNAseq}=0;\mathrm{TPM}<0.50\\ 5\;\; & \mathrm{mut}\;\mathrm{reads}\;\mathrm{RNAseq}=0;\mathrm{wt}\;\mathrm{reads}\;\mathrm{RNAseq}>0;\mathrm{TPM}<0.50 \end{array}$$

### 2.4. GAd Vector Production

To generate the adeno viral vectors (Ad), the selected candidate neoantigens were joined head to tail to generate artificial poly-epitope proteins. A signal peptide was added at N-Term, corresponding to aa 1–29 of the human TPA (tissue plasminogen activator) protein (NP_000921.1). The resulting transgenes were synthesized by GeneART (Thermo Fisher Scientifics) and then transferred into the genome of a Gorilla Adenoviral vector (serotype group C) deleted in E1, E3, and E4 regions and carrying Ad5 E4 ORF6. The resulting recombinant vectors were produced by transfection of adherent M9 cells and amplification in suspension M9 cells. Vectors were then purified from infected cells by Vivapure Adenopack 20 RT (Sartorius).

### 2.5. Mice

Six-week-old female C57BL/6 mice were purchased from Envigo. All day-to-day care was performed by trained mouse house staff at Plaisant, Castel Romano. All experimental procedures were approved by the Italian Ministry of Health and were carried out in accordance with the applicable Italian laws (D.L.vo 26/14 and following amendments), the Institutional Animal Care and ethic Committee of Allevamenti Plaisant SRL.

### 2.6. In Vivo Tumor Growth

2 × 10^5^ MC38 cells were s.c. injected into the lower right flank. Before the start of treatments (day 0), animals were randomized (tumor size average per group 70–100 mm^3^). Mice were sacrificed as soon as signs of distress or a tumor volume above 2000 mm^3^ occurred. Tumor growth was measured using digital caliper every 3–4 days. Tumor volume was calculated using the following formula: 0.5 × length × width^2^, where the length was the longer dimension.

### 2.7. In Vivo Treatments

Vaccine was administered via intramuscular injections in the quadriceps in a volume of 50 μL per side at the dose of 5 × 10^8^ vp. For efficacy studies, α-mPD1 (BioXcell, clone RMP114, Cat. Number: BE0146) was administered twice a week until day 16 post treatment start. To deplete T-cell subsets, α -mCD8 (BioXcell, clone YTS169.4, Cat. Number: BE0117) was administered.

### 2.8. Ex Vivo Immune Analysis

Spleens were harvested 3 weeks post immunization and ex vivo IFN-γ ELISpot was performed as described previously [22].

## 3. Results

### 3.1. VENUS RSUM Score Provides a Ranked List of Neo-Peptides Ordered According to a Balanced Three-Parameter Score

VENUS RSUM score utilizes DNA and RNA sequencing data from a patient’s tumor biopsy/healthy tissue to identify mutations in the tumor. Both SNVs and indels generating an FSP are analysed.

**Figure 1:**
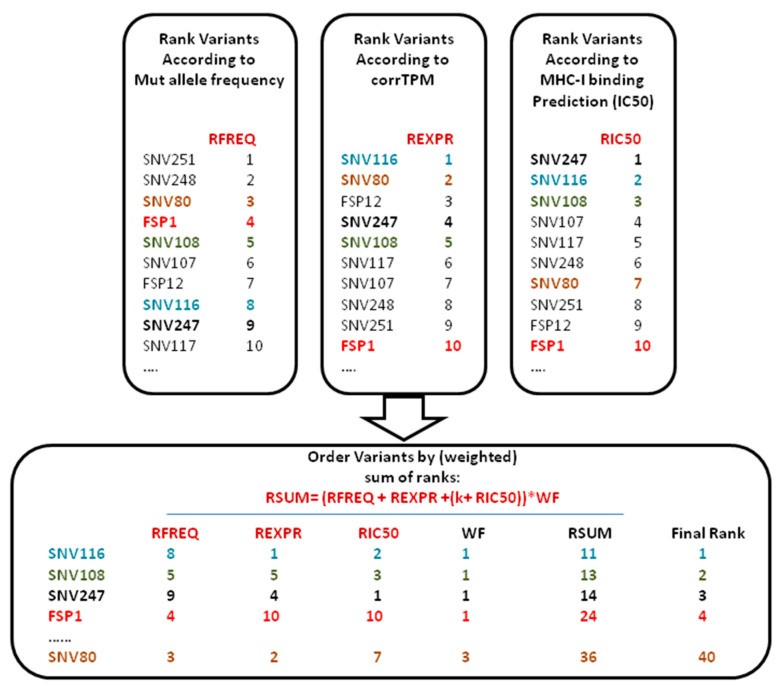
Schematic description of the vaccine-encoded neoantigens unrestricted selection (VENUS) RSUM prioritization method: Schematic description of the ranking procedure applied with VENUS RSUM score. The mutations-encoded neo-peptides are ranked independently three times using three different parameters. For each neo-peptide, the individual ranks are summed and corrected according to weighting factors (k; WF; details in methods) that penalize neo-peptides with a predicted IC50 > 1000 nM (k) and mutations that fall in regions with low read coverage or within not expressed genes according to the next generation sequencing (NGS) mRNA transcriptome data (WF).

The method firstly assigns a score to the following three parameters: allele frequency of the mutations, abundance of the transcripts carrying the mutation, and the likelihood of the generated neo-peptide to bind the patient’s MHC class-I molecules. Then, a final score is assigned to each neo-peptide by summing up the individual scores of the three parameters and applying correction factors (Figure 1 and methods). The characteristic feature of VENUS RSUM is that each parameter independently contributes to the final score thus leading to a final rank for each neo-peptide balanced for the fitness of each single parameter.

To test the performance of the VENUS RSUM method, we analysed available public datasets with complete NGS raw data (healthy/tumor exome and tumor transcriptome) from biopsies of nine cancer patients in which neoantigens inducing spontaneously a T cell response were experimentally verified [11,23,24].

For each patient, we simulated the selection of neoantigens for a putative therapeutic vaccine by first determining an overall list of neo-peptides originating from SNVs or indels (details in methods). The patient-specific mutation lists were then ranked by VENUS (Supplementary Table S1). In total, we identified 2691 potential neoantigens (median of 267 neo-peptides per patient) derived from 1693 somatic mutations (Supplementary Table S1). The discrepancy between the number of potential neoantigens and the total number of detected somatic mutations is due to the detection of few indels encoding FSPs in almost all of the analysed samples. In fact, FSPs, once processed by the MHC presenting machinery, can give rise to multiple smaller peptides, with different likelihoods to be exposed to the immune system. Therefore, all the possible 25 mer peptides derived from the same FSP are ranked independently (details in methods) by the VENUS system.

### 3.2. Venus RSUM Score Captures Validated Neoantigens Eliciting an Immune Response in Humans

To verify the predictive value of VENUS, we then determined the position of neoantigens eliciting an experimentally verified T cell response within the ranked list of neopeptides detected in each patient.

The dataset contains 20 experimentally validated neoantigens (1–4 epitopes per patient; Table 1), for which a specific T cell was identified in the tumor infiltrating lymphocytes (TILs). Therefore, a successful prioritization implies the capability of VENUS to efficiently rank mutations that have been able to prime T cells [11,23,24].

All experimentally validated neoantigens from the validation datasets were ranked among the top 25% neo-peptides for each patient (median top 5%; Table 1).

**Figure 2:**
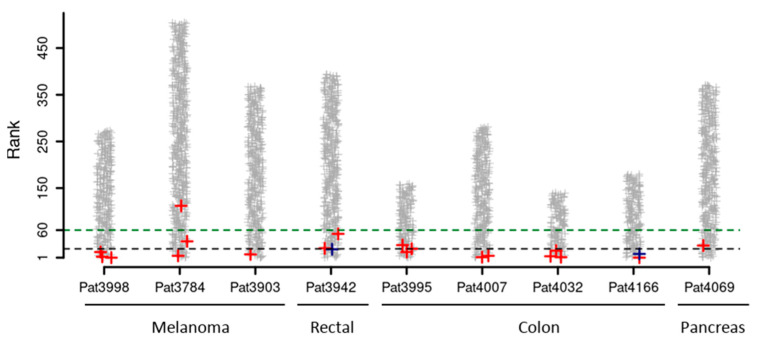
VENUS ranking of experimentally validated neoantigens: Ranking of 20 experimentally validated neoantigens according to VENUS RSUM score. Better ranks correspond to lower values on the y-axis. Grey crosses represent all neo-peptides generated by somatic mutations in each patient. Experimentally validated CD8+ and CD4+ reactivities are depicted in red and blue, respectively. Horizontal lines indicate thresholds for the top 20 (black) and top 60 (green) neoantigens.

As neoantigen-specific immunotherapies are often limited, for practical and/or technical reasons, by the number of neoantigens that can be targeted, we determined the number of experimentally validated neoantigens that fell within the top 20 or top 60 ranked neo-peptides for each patient. The results show that 70% (14 out of 20) and 95% (19 out of 20) of experimentally validated neoantigens would have been targeted by vaccination platforms targeting the 20 or 60 top VENUS ranked neo-peptides, respectively (Figure 2). Interestingly, two validated CD4 T-cell epitopes are captured within the top 20 selection even if VENUS ranks neoantigens only on the basis of MHC-I binding predictions. In this context, the expression level determined as corrected TPM (corrTPM) by VENUS was shown to be very impactful as a single parameter. In fact, the corrTPM looks at the RNA value of the entire mutated gene as well as the value of the small region containing the mutations highlighting the importance to consider both values to determine the expected abundance of the mRNA carrying the mutation.

**Figure 3:**
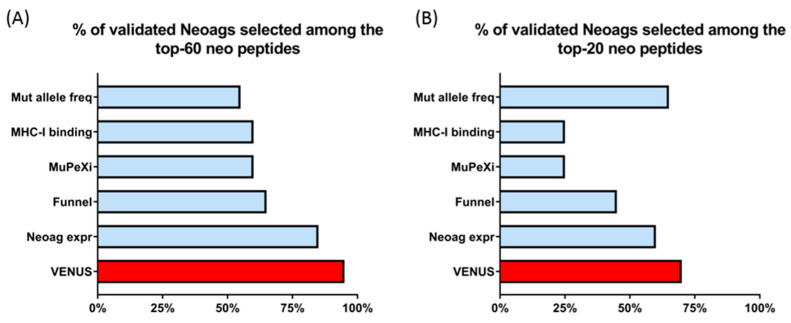
Comparison of VENUS performance against alternative methods that include less parameters. Light blue bars represent the percentage of validated neoantigens included in the top 60 (**A**) or top 20 (**B**) selection performed using the indicated alternative single or combination of parameters. For comparison, the percentage of validated neoantigens included within the top 60 (**A**) or 20 (**B**) neoantigens by VENUS. Funnel filtering is performed by retaining only the neo-peptides predicted as major histocompatibility complex (MHC)-I binders (9 mer; predicted IC50 ≤ 500 nM) and encoded by an expressed gene (transcripts for million (TPM) ≥ 0.50).

We then tested different alternative ways of selecting the top 60 neo-peptides for each patient. All the combinations tested had a reduced capability of selecting validated neoantigens compared with VENUS (Figure 3). Notably, the application of a filter applying fixed thresholds for MHC-I prediction and expression funnel results in a poor performance with only 65% (13 out of 20) of the validated neoantigens selected.

We also compared the performance of VENUS with MuPeXi, a publicly available neoantigen prioritization tool that applies a sigmoidal logistic function to rank neoantigens on the basis of peptide-HLA binding affinity and includes additional features such as the sequence similarity to non-mutated self-proteins [25]. Only 5 (25%) and 12 (60%) experimentally validated neoantigens were ranked in top 20 and 60 selection obtained with MuPeXi, respectively (Figure 3A,B).

### 3.3. Generation of Viral Vectors Targeting the Best Neoantigens Ranked by VENUS in MC38 Tumour Model

**Figure 4:**
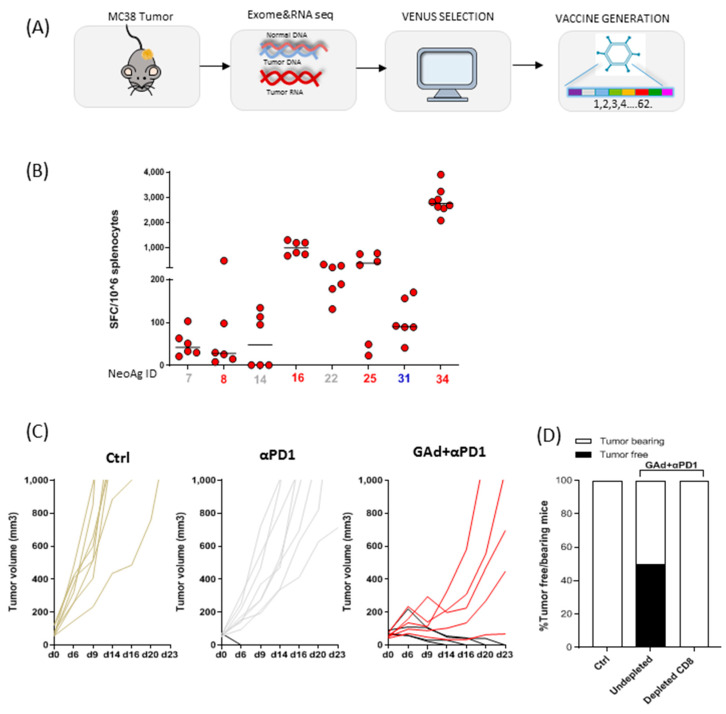
Vaccination with VENUS-identified neoantigens encoded in a GAd vector is effective in the established MC38 tumor model in combination with anti-PD1. (**A**) Schematic of the approach used to identify MC38 tumor specific mutations and generation of the vaccine. (**B**) In vivo immunogenicity of GAd-MC38-62. T-cell responses were measured by IFN-γ ELISpot on splenocytes of naive mice 3 weeks post immunization with 5 × 10^8^ vp of GAd-MC38-62. Responses against the eight immunogenic neoantigens (nAgs) are shown. Neoantigens IDs inducing CD8+ or CD4+ T-cell responses are indicated in red and blue, respectively. Data are representative of two independent experiments. (**C**) Mice were inoculated s.c. with MC38 cells. One week later, animals were randomized according to tumor volume and treated with anti-PD1 alone or in combination with GAd-MC38-62. Vaccine was administered at day 0 (i.m.) following randomization, whereas anti-PD1 was given twice per week until day 17 (i.p.). Tumor growth over time is shown for individual mice. Black curves indicate responder mice showing a complete response post treatment. (**D**) Frequency of tumor free (black) and tumor bearing mice (white) upon treatment with GAd-MC38-62 and anti-PD1 depleted for CD8+ T cells or undepleted.

In order to validate the VENUS method in vivo in the context of vaccination, we used a murine model, namely the MC38 colon cancer model. Tumors derived from the MC38 cell line grown in vivo were resected from C57BL/6 mice and sequenced. Exomeseq and RNAseq data were analysed to identify and rank tumor mutations (Figure 4A).

The adenoviral vector (Ad) platform has the unique feature of encoding very long antigens (up to 2000 amino acids). The ability of Ad vectors to accommodate large gene inserts, and thus target many neoantigens, makes them an ideal platform for cancer vaccine immunotherapy. In the present experiments, this high capacity allowed for encoding 62 mutated peptides selected out of 3605 potential neoantigens by the VENUS RSUM score (Supplementary Table S1).

The 62 potential neoantigens were joined head to tail to generate an artificial transgene that was then cloned into a non-human Great Apes-derived Adenovirus (GAd) vector (GAd-MC38-62). Only 3 (AATF_A500T; CPNE1_D302Y; DPAGT1_V213L) out of the 62 selected candidate neoantigens correspond to sequences previously described in this model [26].

### 3.4. Vaccination with VENUS-Identified Neoantigens Is Effective in Eradicating Large MC38 Tumours in Combination with Anti-PD1

A single intramuscular injection of GAd-MC38-62 vector in naïve mice elicited a strong T cell immunity as measured by ex-vivo IFN-γ ELISpot, with eight of the predicted neoantigens being immunogenic (Figure 4B). Among the most potent immunogenic neoantigens, four induced CD8+ T cells and one CD4+ T cell (Supplementary Figure S2) were identified by intracellular cytokine staining (ICS), confirming that CD4 neoantigens are also captured by the VENUS method.

Previously, we have demonstrated that immunization with a GAd vector encoding neoantigens is effective in eradicating large tumors in combination with anti-PD1 [22].

Efficacy of the GAd-MC38-62 cancer vaccine was thus evaluated in the MC38 mouse model in an aggressive tumor setting. MC38 tumoral cells were inoculated subcutaneously in mice and 8 days post inoculum, once tumors reached a mass volume of 70–100 mm^3^, the GAd-MC38-62 vaccine was administered. In this setting, large tumors are already present at the time of vaccine administration and the vaccine treatment alone is not effective, but requires combination with anti-PD1 treatment for tumor eradication as previously demonstrated [22]. While anti-PD1 alone was effective only in 10% of treated mice, the combined treatment of GAd and anti-PD1 induced tumor eradication in 45% of treated animals (Figure 4C). Vaccine efficacy was shown to be due to the induction of CD8 T cells, given the fact that selective depletion of CD8+ T cells completely abrogated the antitumor effect (Figure 4D). Therefore, the VENUS approach selects neoantigens effective for a therapeutic vaccination approach.

## 4. Discussion

The identification of the best neoantigens to be targeted by a personalized therapeutic cancer vaccine is still a challenging task limited by (a) the accuracy of existing in silico methods and/or (b) the time needed to potentially test all experimentally candidate neoantigens [27].

Here, we demonstrate that the VENUS algorithm can successfully select validated neoantigens inducing effective T-cell mediated immune responses. By testing different prioritization strategies, we have demonstrated that the prediction of neoantigens inducing an immune response in cancer patients is poorly related to single properties like high expression or the high predicted likelihood of binding to MHC class I, but instead requires the combination of multiple parameters to reach an improved predictive power.

One advantage of VENUS is that the score assigned to each neopeptide includes three independent distinct features combining the abundance and expression across the tumor cells of the mutated peptide with the likelihood of being presented by MHC-I. The way in which these features are combined and weighted generates a balanced score that well represents the population of neoantigens presented to T cells, while reducing the chance to include potentially irrelevant mutated peptides. By applying a selection strategy on a human validation datasets, we demonstrated the ability of the VENUS method to capture 95% of validated neoantigens within the top 60 ranked. Moreover, we demonstrated that VENUS out-performed other publicly available methods including one applying expression and MHC-I likelihood of binding in a sequential way.

We acknowledge that one limitation of VENUS is the absence class II HLA binding predictions. We decided to focus only on class-I, because CD8+ T cell responses to neoantigens have been linked to clinical efficacy of immune checkpoint inhibition [28,29]. Furthermore, the accuracy of algorithms to predict class II bound CD4 peptides is still low compared with the accuracy obtainable by class I methods [30,31]. However, despite the formal absence of class II MHC binding predictions, the two validated CD4 neoantigens from two human cancer patients, one for each patient, were correctly ranked at the top of neoantigens selected by VENUS. Focusing only on class I predictions thus does not exclude the possibility to include neoantigens that are recognized by HLA class II for which the selection is based only on the other two weighted parameters.

To demonstrate that VENUS accurately identifies neoantigens to be used in an effective therapeutic vaccine, we validated VENUS-selected epitopes in the MC38 mouse model. Previous data documented the ability of the Adenovector platform to efficiently deliver antigenic proteins up to 2000 aa long as well as being suitable to encode for many neoantigens joined one after the other in an artificial gene [32,33]. Based on the analysis of human data, we decided to target at least 60 mutated peptides in the vaccine to increase the chance of including effective neoantigens. Several mechanisms allow the tumor to evade T-cell attack and, in this complex scenario, the ability of GAd vectors to allow targeting many neoantigens offers the advantage to potentially overcome the issue of tumor heterogeneity and escape through immunoediting. To validate the strategy, the top 62 neoantigens scored by Venus in the MC38 tumor cell line were delivered via GAd vaccine vector. To our knowledge, 62 represents the highest number of tumor neoantigens targeted by a personalized cancer vaccine. Vaccination induced in mice a potent T cell response against the number of encoded neoantigens comprising antigen-specific IFN-γ secreting CD8+ and CD4+ T cells. Importantly, in line with our previous findings, therapeutic vaccination with a GAd vector encoding neoantigens showed synergy with PD1 blockade, resulting in eradication of large established tumors in about 50% of mice treated with the combo versus 15% in those treated with anti-PD1 monotherapy. Interestingly, all but three of the peptides selected by VENUS have not been reported in previous studies based on mass spectrometry analysis on the same murine tumor model. This finding highlights the limit of MS spectra-based methods capturing only a subset of the effective neoantigens [34].

For the development of personalized genetic vaccines, it is very important to produce viral vectors targeting the “private” neoantigens owned by a patient in the shortest timeframe since biopsy collection. In this context, an added value of VENUS RSUM score is that the lack of fixed thresholds for selecting the neo-peptides to be targeted by genetic vaccines eases the design steps and contributes to speeding up the entire manufacturing process.

The VENUS algorithm is now in use to select up to 60 patients specific neoantigens in a Phase 1b personalized vaccine trial (NOUS-PEV-01) in NSCLC and melanoma metastatic patients. The vaccination regimen is based on heterologous prime boost with GAd and MVA viral vectors used in combination with anti-PD1 check point inhibitor.

## 5. Conclusions

In conclusion, we developed an innovative score methodology, called VENUS RSUM, to prioritize tumor specific neo-peptides with high probability to induce a T-cell mediated immune response against cancer cells. The efficacy of our tool was demonstrated in silico by reanalyzing NGS data of patients with different solid tumor types for whom experimental data were available. Based on the analysis of this human dataset, we developed a viral vector platform able to target at least 60 mutated peptides to maximize the chance of including effective neoantigens and potentially overcoming the issue of tumor heterogeneity and escape through immunoediting. To further validate the strategy, the top 62 neoantigens scored by VENUS in the MC38 tumor cell line were delivered via GAd vaccine vector. Sixty-two represents the highest number of tumor neoantigens targeted by a personalized cancer vaccine, and the vaccination induced in mice a potent T cell response in mice against a number of encoded neoantigens comprising antigen-specific IFN-γ secreting CD8+ and CD4+ T cells.

## Figures and Tables

**Table 1:** Position of experimentally validated neoantigens in patient-specific lists of ranked according to VENUS score. For each patient, the total number of mutation-derived neo-peptides, the position of the neoantigens with validated T-cell reactivities in the protein, and the position of the neoantigen within the VENUS ranked list are reported, and for each neoantigen, the percentage of neo-peptides that were ranked with a better score is reported.

Tumor Type	Patient ID	Total Detected Neo-Peptides	Experimentally Validated Neoantigens	Position in VENUS Ranked List	Percentage of Neo-Peptides Ranked Better
Melanoma	3998	268	MAGEA6_E168K	1	0%
			PDS5A_H1007Y	3	1%
			MED13_P1691S	13	5%
	3784	494	FLNA_R2049C	5	1%
			SON_R1927C	36	7%
			KIF16B_L1009P	110	22%
	3903	435	KIF1BP_P246S	8	2%
Rectal	3942	396	GPD2_E426K	19	5%
			NUP98_A359D	21	5%
			KARS_D328H	58	15%
Colon	3995	138	RNF213_N1702S	13	9%
			TUBGCP2_P265L	20	14%
			KRAS_G12D	28	20%
	4007	262	SKIV2L_R653H	1	0%
	4032	136	API5_R243Q	2	1%
			PHLPP1_G566E	4	3%
			RNF10_E572K	16	12%
	4166	180	NPLOC_G1473V	1	0%
			SUN1_A127T	9	5%
Pancreas	4069	371	ZFYVE27_R6H	41	11%

## Data Availability

Mouse and human NGS data are public and available in Bioproject NCBI portal (IDs PRJNA543001, PRJNA298310, PRJNA298330, PRJNA298376, PRJNA298330.

## Supplementary Materials

The following are available online at https://www.mdpi.com/article/10.3390/vaccines9080880/s1. Figure S1: Tailoring procedure applied to FSPs: Figure S2: Vaccine-encoded immunogenic neoantigens induce CD8+ and CD4+ T cell responses. TableS1 list of somatic mutations encoding neo-peptides ranked by VENUS.

## Abbreviations

CPIs	Check point inhibitors
NGS	Next generation sequencing
SNVs	Single nucleotide variants
FSP	Frame shift peptide
MHC	Major histocompatibility complex
VENUS	Vaccine-encoded neoantigens unrestricted selection
MF	Mutation allele frequency
TPM	Transcripts for million
corrTPM	corrected transcripts for million
TPA	Tissue plasminogen activator
TetO	Tet operator
Ad	Adenoviral vector
GAd	Great apes-derived adenovirus
TIL	Tumor infiltrating lymphocytes
s.c	Subcutaneous
i.v	Intravenous
i.m	Intramuscle
