# Supplementary material for: VENUS, a Novel Selection Approach to Improve the Accuracy of Neoantigens’ Prediction
Source: Vaccines (Basel). 2021 Aug 9;9(8):880. doi: 10.3390/vaccines9080880 (PMC8402534; doi:10.3390/vaccines9080880)
Supplement: Supplementary file 1 [file vaccines-09-00880-s001.zip › vaccines-1268365 supplementary .pdf]

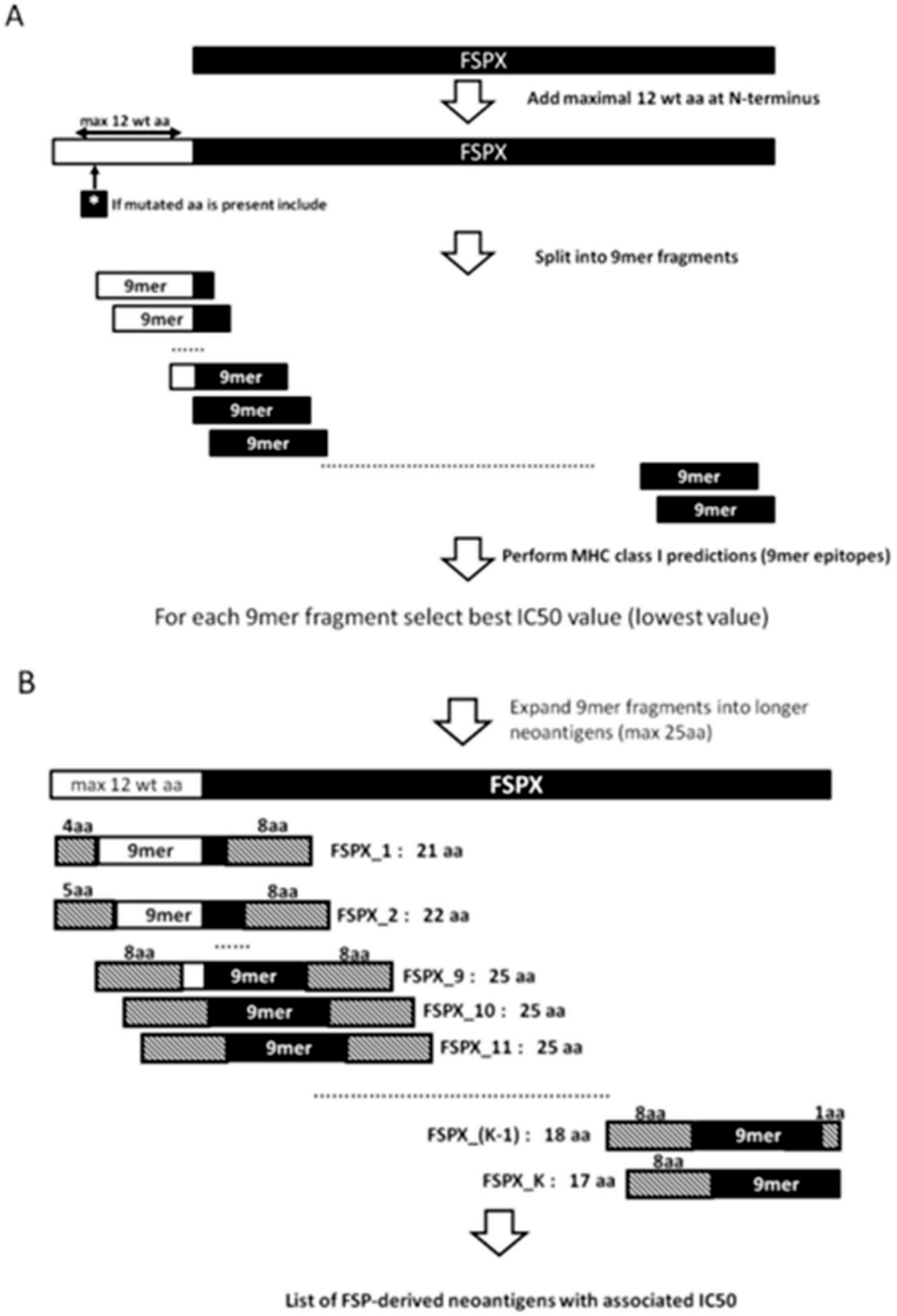

Figure 1. "Tailoring" procedure applied to FSPs: In figure is schematized the procedure followed by the algorithm to split and rank the neo peptides derived from FSPs A) Whenever possible, to the N terminus of each long FSP sequence are added 12 wild type aminoacids. A windows of 9 aminoacids length is slided along the entire FSP to generate all the possible 9 mers peptides. For each peptide are performed MHC-I binding predictions and in case of multiple binding alleles is selected the best prediction. B) Each 9-mer peptide is then extended into a 25mer by adding 8 amino acids to both ends. All FSP-derived 25mers are finally added to the list of SNV-derived 25mer and subjected to the ranking procedure.

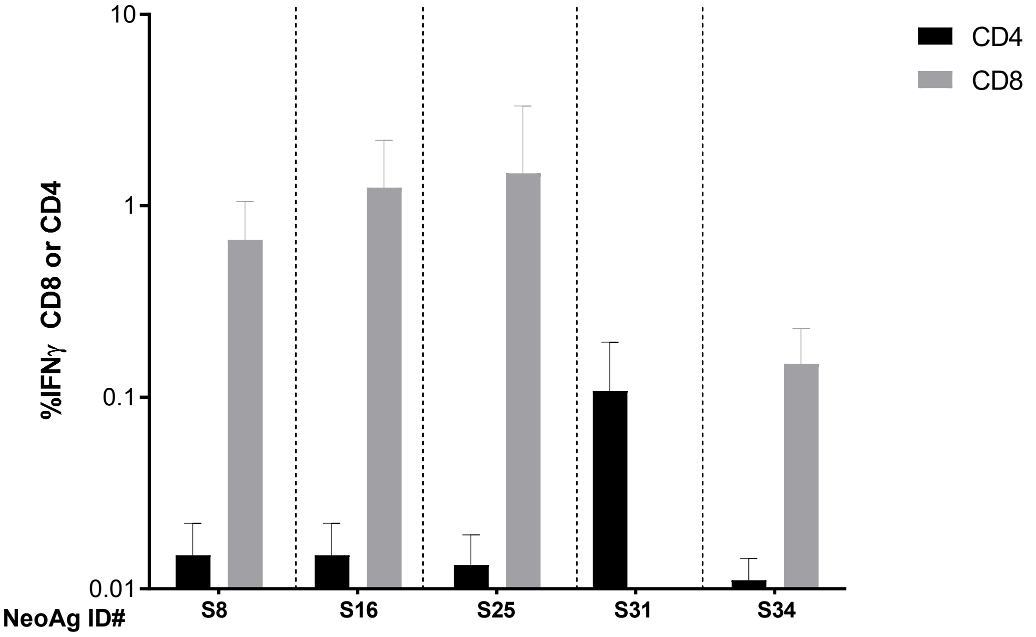

Figure S2: Vaccine-encoded immunogenic neoantigens induce $CD8^+$ and $CD4^+$ T cell responses. Characterization of T cell subtypes induced by immunogenic nAgs after GAd-MC38-62 vaccination evaluated by intracellular cytokine staining (ICS) and fluorescence-activated cell sorting (FACS). 3 weeks post vaccination splenocytes were stimulated with individual immunogenic peptides. Data (means ± SEM) show the percentages of IFN-γ + CD4+ and CD8+ T cells and are representative of 2 experiments.
